# Efficacy of Chinese herbal medicine for stroke modifiable risk factors: a systematic review

**DOI:** 10.1186/s13020-017-0146-9

**Published:** 2017-09-05

**Authors:** Wenbo Peng, Romy Lauche, Caleb Ferguson, Jane Frawley, Jon Adams, David Sibbritt

**Affiliations:** 1 Australian Research Centre in Complementary and Integrative Medicine (ARCCIM), University of Technology Sydney, Ultimo, NSW Australia; 2 Centre for Cardiovascular and Chronic Care, University of Technology Sydney, Ultimo, NSW Australia; 3 Australian Research Centre in Complementary and Integrative Medicine (ARCCIM), Faculty of Health, University of Technology Sydney, Level 8, Building 10, 235-253 Jones St, Ultimo, NSW 2007 Australia

**Keywords:** Chinese herbal medicine, Stroke, Risk factor, Prevention

## Abstract

**Background:**

The vast majority of stroke burden is attributable to its modifiable risk factors. This paper aimed to systematically summarise the evidence of Chinese herbal medicine (CHM) interventions on stroke modifiable risk factors for stroke prevention.

**Methods:**

A literature search was conducted via the MEDLINE, CINAHL/EBSCO, SCOPUS, and Cochrane Database from 1996 to 2016. Randomised controlled trials or cross-over studies were included. Risk of bias was assessed according to the Cochrane Risk of Bias tool.

**Results:**

A total of 46 trials (6895 participants) were identified regarding the use of CHM interventions in the management of stroke risk factors, including 12 trials for hypertension, 10 trials for diabetes, eight trials for hyperlipidemia, seven trials for impaired glucose tolerance, three trials for obesity, and six trials for combined risk factors. Amongst the included trials with diverse study design, an intervention of CHM as a supplement to biomedicine and/or a lifestyle intervention was found to be more effective in lowering blood pressure, decreasing blood glucose level, helping impaired glucose tolerance reverse to normal, and/or reducing body weight compared to CHM monotherapy. While no trial reported deaths amongst the CHM groups, some papers do report moderate adverse effects associated with CHM use. However, the findings of such beneficial effects of CHM should be interpreted with caution due to the heterogeneous set of complex CHM studied, the various control interventions employed, the use of different participants’ inclusion criteria, and low methodological quality across the published studies. The risk of bias of trials identified was largely unclear in the domains of selection bias and detection bias across the included studies.

**Conclusion:**

This study showed substantial evidence of varied CHM interventions improving the stroke modifiable risk factors. More rigorous research examining the use of CHM products for sole or multiple major stroke risk factors are warranted.

## Background

Stroke is the second foremost cause of mortality and a leading cause of serious disability worldwide [1]. The incidence of stroke continues to rise due to societal and lifestyle changes and an aging population [2]. More than 90% of the stroke burden is attributable to its modifiable risk factors such as high blood pressure, high fasting plasma glucose, and high total cholesterol [3]. These stroke risk factors are strongly inter-related and some of them are simultaneous shown as a combined risk factor in people with stroke with higher risk [4, 5]. Previous research has clearly demonstrated the benefits of treating risk factors such as hypertension, diabetes, hyperlipidemia, obesity, atrial fibrillation, or transient ischaemic attack (TIA) for reducing the prevalence of primary stroke [6, 7]. The treatments of major stroke modifiable risk factors are therefore crucial for informing stroke prevention strategies and helping achieve improved quality of life of people with those risk factors and lowered associated health care costs [3].

Chinese herbal medicine (CHM)—therapies and products made from any part of medicinal plants (e.g. leaves and roots) and some non-herb based components (e.g. shells and powdered fossil) [8]—has a history of more than 2500 years with a unique theory of diagnosis and treatment, and is considered a modality of complementary medicine in Western countries [9]. CHM has been increasingly used for a wide range of chronic diseases in China and elsewhere in the form of raw plant materials, powers, capsules, tablets and/or liquids [9–11].

Chinese herbal medicine is a field of health care that may offer potential for addressing related risk factors of stroke [12–14]. Many CHM interventions have long been used for the treatments of some stroke risk factors as individual diseases such as Type 2 diabetes [15], hypertension [8] and obesity [16]. However, the research evidence as to whether specific CHM therapies or products may be effective in reducing each individual or mixed major risk factors of stroke remains unclear. The aim of this systematic review is to assess and summarize the efficacy and safety of all relevant CHM interventions for people at greatest risk(s) of stroke.

## Methods

### Search strategy

Four key bibliographic databases—MEDLINE, CINAHL/EBSCO, SCOPUS, and Cochrane Database of Systematic Reviews—were searched in the systematic review. This review was designed and conducted in accordance with PRISMA (Preferred Reporting Items for Systematic Reviews and Meta-Analyses) guidelines. The stroke modifiable risk factors identified in this systematic review refer to high blood pressure (hypertension), high cholesterol (hyperlipidemia), irregular pulse (atrial fibrillation), TIA, high blood glucose (diabetes and impaired glucose tolerance (IGT), and overweight (obesity). The literature search employed keyword and MeSH term searches for terms relevant to ‘CHM’ and terms regarding stroke risk factors (Table 1). The combination of the search results of CHM and stroke risk factors were identified for screening. To obtain all relevant articles, reference lists of published review papers were also reviewed via Google Scholar.

**Table 1:** Search terms for the systematic review

Chinese herbal medicine	Chinese herbal medicine [MeSH Term & Keyword] OR Chinese medicine [MeSH Term & Keyword] OR Chinese herb* [Title/Abstract] OR Chinese herbal [Title/Abstract]	
AND		
Stroke risk factors	High blood pressure	Hypertension [MeSH Term & Keyword] OR Blood pressure [MeSH Terms & Keyword] OR Hypertens* [Title/Abstract] OR Prehypertens* [Title/Abstract] OR Systolic [Title/Abstract] OR Diastolic [Title/Abstract] OR
	High cholesterol	Cholesterol [MeSH Term & Keyword] OR Triglycerides [MeSH Term & Keyword] OR Dyslipidemia [MeSH Term & Keyword] OR Epicholesterol [Title/Abstract] OR HDL [Title/Abstract] OR LDL [Title/Abstract] OR Triglyceride* [Title/Abstract] OR Hyperlipidem* [Title/Abstract] OR Lipidem* [Title/Abstract] OR
	Irregular pulse	Cardiac arrhythmias [MeSH Terms & Keyword] OR Atrial fibrillation [MeSH Terms & Keyword] OR Dysrhythmia* [Title/Abstract] OR Cardiac arrhythmia* [Title/Abstract] OR
	Transient ischaemic attack	Transient ischaemic attack [MeSH Terms & Keyword] OR Transient ischaemic attack* [Title/Abstract] OR
	High blood glucose	Diabetes [MeSH Terms & Keyword] OR Mellitus [MeSH Terms & Keyword] OR Impaired glucose tolerance [MeSH Terms & Keyword] OR Diabet* [Title/Abstract] OR NIDDM [Title/Abstract] OR IDDM [Title/Abstract] OR T2DM [Title/Abstract] OR *insulin* [Title/Abstract] OR Glucose [Title/Abstract] OR
	Overweight	Obesity [MeSH Terms & Keyword] OR Overweight [MeSH Terms & Keyword] OR Metabolic syndrome [MeSH Terms & Keyword] OR Obes* [Title/Abstract] OR Adiposity [Title/Abstract] OR Adipos* [Title/Abstract]

* Truncation, refering to all records that have those letters with any ending

### Study selection

The inclusion criteria of literature in the systematic review were: peer-reviewed English-language journal articles focusing upon randomized controlled trials (RCTs) or cross-over studies published in the past 20 years (1996–2016), and articles reporting primary data findings examining the efficacy and safety of any type of CHM interventions (e.g. decoction, capsule, granule, power) on one or more major modifiable risk factors of stroke. Exclusion criteria were (1) published RCT protocols of this research area; (2) quasi- or pseudo-RCTs (3) studies focusing upon the efficacy and safety of CHM for treating stroke or post-stroke symptoms; (4) studies focusing upon the efficacy and safety of CHM for treating the complications of the stroke risk factors; (5) conference abstracts; and (6) publications without abstracts.

### Data extraction

Titles and abstracts of all citations identified in the initial search were imported to Endnote (Version X7) and duplicates removed. Two of the authors screened all the titles/abstracts to identify articles meeting the inclusion and exclusion criteria independently. When consensus was not reached, the full texts of these unclear papers were retrieved and assessed by these two authors. Disagreements were discussed with a third author.

Data were extracted into a pre-determined table (Table 2) and checked for coverage and accuracy by two of the authors. Any differences in data extraction and interpretation were resolved through discussion amongst all authors. Table 2 includes detailed information on study recruitment, participant characteristics, intervention groups, results of primary outcome measures, study limitations, and CHM safety.

**Table 2:** Characteristics of the included studies

Author Country Study period	Stroke risk factor	Participants	Intervention groups		Results	Side effects	Limitations
			Treatment group(s)	Control group(s)			
Lin et al. [18] China Sep 2001–Sep 2002	(Primary) Hypertension	*Sample size* n = 102 *CHM group* n = 52; 41 males and 11 females; mean age: 55 years *Control group* n = 50; 41 males and 9 females; mean age: 54 years *Inclusion criteria* SBP: 140–179 mmHg or DBP: 90–109 mmHg; TCM diagnosed for hyperactivity of the liver-yang syndrome	*Tianma gouteng decoction* 150 ml/time, twice daily, 4 weeks *Formulas* Tianma, Niuxi, Sangjisheng, Yimucao, Yejiaoteng, Huangqi, et al.	*Nitrendipine* 10 mg/time, 3 times daily, 4 weeks	*Baseline balance* Yes Significantly decreased SBP and DBP of both CHM and control groups before and after treatment, without significant difference between these two groups after treatment	No side effects	N/A
Li [19] China No information on study period	(Primary) Hypertension	*Sample size* n = 72 *CHM group* n = 46; 18 males and 28 females; mean age: 54 years *Control group*: n = 26; 11 males and 15 females; mean age: 53 years Both groups have cases with coronary heart disease, hyperlipemia, and diabetes *Inclusion criteria* SBP: 140–179 mmHg or DBP: 90–109 mmHg; TCM diagnosed for flaming-up of the liver-fire syndrome	During the intervention, no other drugs		*Baseline balance* Yes An effective rate (return to the normal range of BP or ≥20 mmHg but not in the normal range) at 60.9% of hypertension in the CHM group and 15.4% in the control group; Significantly decreased cholesterol, TG, blood sugar of the CHM group before and after treatment, without significant difference compared to the control group after treatment	CHM group: Vomiting and distension (n = 1); Slight abdominal pain and diarrhea (n = 3)	N/A
			*Huanglian fire-purging mixture* 30 ml/time, twice daily, 4 weeks *Formulas* Huanglian, Gouteng, Zexie, Luhui	*Niuhuang Bolus* 1–2 bolus/time, 2–3 times daily, 4 weeks			
Ye et al. [20] China Feb 2004–Dec 2004	(Primary) Hypertension	*Sample size* n = 55 *CHM group* n = 28 *Control group* n = 27 *Inclusion criteria* SBP: 140–179 mmHg or DBP: 90–109 mmHg; normal LDL-C level; currently no antihypertensive medications or using antihypertensive medications for at least 6 months before screening	*Xuezhikang with Nifedipine* (20 mg/time, twice daily) 1200 mg daily, 72 weeks *Formulas* Red yeast rice	*Placebo with Nifedipine* (20 mg/time, twice daily) 1200 mg daily, 72 weeks	*Baseline balance* Yes No significant differences in BP between the CHM and placebo groups after treatment; 92.8% of the CHM group and 88.9% of the placebo group reached the target BP (<140/90 mmHg)	N/A	N/A
Zhao et al. [21] China No information on study period	(Primary) Hypertension	*Sample size* n = 79 *CHM group* n = 40; 17 males and 23 females; mean age: 52 years *Control group* n = 39; 18 males and 21 females; mean age: 52 years *Inclusion criteria* SBP: 140–159 mmHg or DBP: 90–99 mmHg; no antihypertensive drugs or stopped taking antihypertensive drugs for 2 weeks; TCM diagnosed for stagnation of phlegm, blood stasis and hyperactivity of the liver-yang syndrome; age: 40–60 years	*Yinian Jiangya Yin* 100 ml/time, twice daily, 15 days *Formulas* Gouteng, Shijueming, Yimucao, Guijia, Banxia, Zhike, et al.	*Tianma Gouteng Yin* 100 ml/time, twice daily, 15 days *Formulas* Tianma, Gouteng, Huangqin, Yejiaoteng, Fushen, Duzhong, et al.	*Baseline balance* Yes Significantly decreased SBP and DBP of both CHM and control groups before and after treatment; Significantly decreased SBP and DBP in the CHM group than those in the control group after treatment; The total effective rate at 95.0% of BP control in the CHM group, while 87.2% in the control group	No side effects	N/A
Zhong et al. [22] China Jan 2006–Dec 2008	(Primary) Hypertension	*Sample size* n = 57 *CHM group* n = 31 *Control group* n = 26 *Inclusion criteria* SBP: 140–179 mmHg or DBP: 90–109 mmHg; daytime BP > 135/85 mmHg or nighttime BP > 120/70 mmHg; age: 18 years and older	During the intervention, no antiplatelet or lipid-lowering drugs and other Chinese patent medicines		*Baseline balance* Yes Significantly decreased SBP and DBP in both CHM and control groups before and after treatment, without significant difference between these two groups after treatment	N/A	N/A
			*Jiangya capsule with Nimodipine simulation* (1 capsule simulation/time, 3 times daily) 4 capsules/time, 3 times daily, 4 weeks *Formulas* Dilong, Nuxi, Haizao, Tianma, Chuanxiong	*Control group 1: Integrative medicine* 4 Jiangya capsule with 1 nimodipine capsule 3 times daily, 4 weeks *Control group 2: Western medicine* 4 Jiangya capsule simulation with 1 nimodipine capsule 3 times daily, 4 weeks			
Yang et al. [23] Taiwan Sept 2008–Aug 2009	(Uncontrolled primary) Hypertension	*Sample size* n = 55 *CHM group* n = 30 *Control group* n = 25 *Inclusion criteria* sitting SBP ≥ 140 mmHg or sitting DBP ≥ 90mHg despite the conventional antihypertensive treatment; TCM diagnosed for hyperactivity of the liver-yang syndrome; age: 18–80 years	*Fufang* *Danshen capsule* 1000 mg/time, twice daily, 12 weeks *Formulas* Gegen, Juhua, Danshen, Hongjingtian	*Placebo* 12 weeks	*Baseline balance* Yes BP control rate (SBP < 140 mmHg and DBP < 90 mmHg) at 25.5% in the CHM group and 7.3% in the placebo group; More significant decrease of SBP in the CHM group than that of the placebo group after treatment	Mild side effects (e.g. diarrhea, fatigue, common cold) (CHM: n = 13; Control: n = 15)	Small sample size; Short study period
Tong et al. [24] China Mar 2010–Sep 2010	(Mild to moderate) Hypertension	*Sample size* n = 219 *CHM group* n = 106; 61 males and 45 females; mean age: 52 years *Control group* n = 113; 62 males and 51 females; mean age: 52 years *Inclusion criteria* SBP: 140–180 mmHg or DBP: 90–110 mmHg; age: 18–65 years; WC ≥ 85 cm (male)/80 cm (female); plus one of the following: (1) TG ≥ 1.7 mmol/l or have received antidyslipidemia treatment; (2) HDL-C < 0.9 mmol/l (male)/1.1 mmol/l (female), or have received the related treatment; (3) FPG ≥ 5.6 mmol/l, diagnosed Type 2 diabetes, or have received glycemiccontrol treatment; (4) TCM diagnosed for liver and stomach damp-heat syndrome	*Jiangzhuoqinggan* 170 ml/time, twice daily, 4 weeks *Formulas* Huanglian, Huangbai, Gouteng, Yinyanghuo	*Irbesartan* 150 mg/time, once daily, 4 weeks	*Baseline balance* Yes Significantly decreased BP in both CHM and control groups before and after treatment, without significant difference between these two groups after treatment; More significant decrease of daytime and nighttime SBP and DBP in the CHM group than those in the control group after treatment; Significantly decreased WC in the CHM group before and after treatment	N/A	Short study period; No placebo group; Small sample size
Wu et al. [25] China Jan 2010–May 2012	(Primary) Hypertension	*Sample size* n = 137 *CHM group 1* n = 45; 31 males and 14 females; mean age: 50 years *CHM group 2* n = 47; 33 males and 14 females; mean age: 48 years *Control group* n = 45; 29 males and 16 females; mean age: 48 years *Inclusion criteria* diagnosed primary hypertension for at least 3 months prior to screening; age: 18–75 years; 24 h MBP ≥ 130/80 mmHg, MBP ≥ 135/85 mmHg during waking hours, or MBP ≥ 120/70 mmHg during sleeping hours; or SBP ≥ 140 mmHg and/or DBP ≥ 90 mmHg	*CHM group 1: Bushen Qinggan granule with amlodipine* (5 mg/time, twice daily) Twice daily, 8 weeks *CHM group 2: Bushen Qinggan decoction with amlodipine* (5 mg/time, twice daily) Twice daily, 8 weeks *Formulas* Tianma, Gouteng, Duzhong, Huangqin, Kudingcha	*Placebo with amlodipine* (5 mg/time, twice daily) Twice daily, 8 weeks	*Baseline balance* Yes Significantly decreased BP in all three groups before and after treatment; Significant decrease in the daytime SBP in the CHM group 2 than that in the other two groups after treatment; More significant decrease of BP variability in the two CHM groups than those in the placebo group, without significant difference between these two CHM groups after treatment	N/A	N/A
Li et al. [26] China Jun 2007–Jan 2008	(Isolated systolic) Hypertension	*Sample size* n = 241; 98 males and 143 females; mean age: 67 years *CHM group* n = 80 *Control group 1* n = 76 *Control group 2* n = 85 *Inclusion criteria* diagnosed hypertension; after 1-week elution period, sitting SBP: 140–180 mmHg and sitting DBP < 90 mmHg; age: 60–80 years	During the intervention, no other antihypertensive drugs		*Baseline balance* Yes Significantly decreased SBP in all three groups before and after treatment; More significant decrease of SBP in the control group 1 than that in the CHM group and control group 2, without significant difference between the CHM group and control group 2 after treatment	Stomach discomfort (CHM: n = 2; Control 2: n = 2); Facial flush and dizziness (Control 2: n = 1)	N/A
			*Jiangya capsule with Nimodipine simulation* (1 capsule simulation/time, 3 times daily) 4 capsules/time, 3 times daily, 4 weeks *Formulas* Dilong, Nuxi, Haizao, Tianma, Chuanxiong	*Control group 1: Integrative medicine* 4 Jiangya capsule with 1 nimodipine capsule 3 times daily, 4 weeks *Control group 2: Western medicine* 4 Jiangya capsule simulation with 1 nimodipine capsule 3 times daily, 4 weeks			
Chen et al. [27] China 2006–2010	(Polarized) Hypertension	*Sample size* n = 125 *CHM group* n = 66 *Control group* n = 59 *Inclusion criteria* SBP > 140 mmHg and DBP < 70 mmHg; age: 60 years and older	Diet, exercise, smoking/alcohol advices were provided; no other Western medicine affecting BP		*Baseline balance* Yes Significantly decreased SBP and pulse pressure in the CHM group before and after treatment; Significantly decreased SBP in the control group before and after treatment; No significant difference of DBP between the two CHM capsule groups after treatment	Dizziness and weakness (CHM: n = 5; Control: n = 4); Pretibial edema (CHM: n = 4; Control: n = 4); Facial flushing and headache (CHM: n = 4; Control: n = 4); Severe side effects (Control: n = 21)	N/A
			*Shiyiwei Shenqi capsule or Dengzhan Shengmai capsule with Amlodipine Besylate tablets and* *Irbesartan tablets* 3-5 capsules/time, 2–3 times daily, 6 weeks *Formulas* *Shiyiwei Shenqi capsule*-Danggui, Xixin, Gouqi, Huangqi, Juemingzi, Lurong, et al. *Dengzhan Shengmai capsule*-Wuweizie, Xixin, Ginseng, Maidong	*Amlodipine Besylate tablets and* *Irbesartan tablets* 5 mg/time, once or twice daily, 6 weeks			
Gong et al. [28] China Apr 2007–Apr 2009	Hypertension with cardiac damage	*Sample size* n = 90 *CHM group* n = 32; 19 males and 13 females; mean age: 59 years *Control group 1* n = 30; 18 males and 12 females; mean age: 56 years *Control group 2* n = 28; 15 males and 13 females; mean age: 59 years *Inclusion criteria* SBP ≥ 140 mmHg and/or DBP ≥ 90 mmHg	Co-administered medications: aspirin, β-blockers, calcium antagonists, diuretics		*Baseline balance* Yes Significantly decreased SBP, DBP in all three groups before and after treatment; More significant decrease of SBP, DBP, TO, LVMI in the CHM group and control group 1 than those in the control group 2 after treatment	Nausea and gastric discomfort (CHM: n = 3; Control 1:n = 1; Control 2: n = 2); Skin rash (Control 1: n = 1)	N/A
			*Xuezhikang capsule with Valsartan* (80 mg/time, once daily) 600 mg/time, twice daily, 24 months *Formulas* Red yeast rice	*Control group 1: Valsartan* 80 mg/time, once daily, 24 months *Control group 2: No intervention*			
Xu et al. [29] China Jan 2006–Apr 2006	Hypertension, hypertension with diabetes, hypertension with coronary heart disease	*Sample size* n = 108 *CHM group* n = 55 *Control group* n = 53 Both groups have cases with diabetes and cases with coronary heart disease *Inclusion criteria* SBP > 140 mmHg or DBP > 90 mmHg; age: 40–80 years	*Qian Yang He Ji with antihypertensive angiotensin II receptor blocker therapy* 35 ml/time, twice daily, 6 months *Formulas* Gouteng, Shengdihuang, Jili, Nvzhenzi	*Antihypertensive angiotensin II receptor blocker* No information of usage	*Baseline balance* Yes Significantly decreased SBP, DBP, pulse pressure, cardioankle vascular index of both CHM and control groups before and after treatment; More significant decrease of SBP, DBP, cardioankle vascular index in the CHM group than those in the control group after treatment	CHM group: serious side effects (n = 5)	N/A
Chao et al. [30] China Sep 2006–Nov 2007	Type 2 diabetes	*Sample size* n = 43; age range: 18–70 *Inclusion criteria* newly diagnosed Type 2 diabetes; FPG ≥ 7 mmol/l and/or OGTT 2hPG ≥ 11.1 mmol/l; BMI: 23–35 kg/m^2^ with poor glucose level after a 1-month diet control (i.e., FPG: 7–10 mmol/l); no antidiabetic drugs before	Diet and exercise advices were provided. During the intervention, no antidiabetic medications		*Baseline balance* Yes Significantly decreased FPG, PPG, HbA1c, BMI in the CHM group before and after treatment, without significant difference between these two groups after treatment	Moderate constipation (CHM: n = 2; Placebo: n = 2)	N/A
			*CHM compound* 3 times daily, 3 months *Formulas* Huanglian, Huangqi, Rendongteng	*Placebo* 3 times daily, 3 months			
Ji et al. [31] China Dec 2007–Oct 2008	Type 2 diabetes	*Sample size* n = 627 (1) Drug naïve group; mean age: 54 years *CHM group* n = 153 *Control group* n = 150 (2) Metformin group; mean age: 55 years *CHM group* n = 164 *Control group* n = 160 *Inclusion criteria* diagnosed Type 2 diabetes; age: 21–70 years; BMI: 18–28 or 18–35 kg/m^2^ using metformin at 750 mg/day (or more) for at least 3 months before screening; stable body weight within at least 3 months before screening; FPG: 7.0–13.0 mmol/l and HbA1c >7%	Diet and exercise advices were provided		*Baseline balance* Yes In drug naı¨ve group: Significant 38% lower any hypoglycemia rate and 41% lower mild hypoglycemic episode in the CHM group than those in the control group after treatment; In Metformin group: Significant 24% lower hypoglycemia rate in the CHM group than that in the control group, without significant difference between these two groups in the mild hypoglycemic episode after treatment; In both drug naı¨ve group and Metformin groups, no significant difference of the rate of reducing HbA1c <6.5% between the CHM and control groups	Urinary tract infection; Upper respiratory tract infection; Elevated ALT/AST; Dyslipidemia	N/A
			*Drug naïve group* *Xiaoke pill* 5–30 pills daily (according to FPG level), 48 weeks *Formulas* N/A	*Glibenclamide* 1.25–7.5 mg daily (according to FPG level), 48 weeks			
			*Metformin group*: *Xiaoke pill with Metformin* (250 mg/tablet) 5 tablets daily, 48 weeks *Formulas* N/A	*Glibenclamide with Metformin* 1.25 mg daily, 48 weeks			
Tong et al. [32] China May 2009–Dec 2009	Type 2 diabetes	*Sample size* n = 480 *CHM group* n = 360 *Control group* n = 120 *Inclusion criteria* early diabetic status; BMI ≥ 24 kg/m^2^; HbA1c ≥ 7.0%; FPG: 7.0–13.9 mmol/l or 2hPG > 11.1 mmol/l; age: 35–65 years	During the intervention, antihyperlipidemia or antihypertensive drugs remain stable		*Baseline balance* statistically different in HbA1c and 2hPG between groups Significantly decreased HbAlc, FPG, 2hPG and increased HOMA-β in both CHM and placebo groups before and after treatment; Significant higher proportion of the HbA1c reversed to normal (HbA1c ≤ 6.5%) in the CHM group (47.6%) than that in the placebo group (35.5%) after treatment; More significant decrease of HbAlc, FPG, 2hPG, body weight, BMI, WC and increase of HOMA-β in the CHM group than those in the placebo group after treatment	Mild side effects (CHM: n = 24; Placebo: n = 7); Transient slight ALT elevation (CHM: n = 2); Transient slight AST elevation (CHM: n = 2)	Short study period; No follow-up
			*Tang-Min-Ling-Wan* 6 g/time, 3 times daily, 12 weeks *Formulas* Huangqin, Huanglian, Baishao, Chenpi, Dahuang	*Placebo* 6 g/time, 3 times daily, 12 weeks			
Tu et al. [33] China No information on study period	Type 2 diabetes	*Sample size* n = 80 *CHM group* n = 41 *Control group* n = 39 *Inclusion criteria* diagnosed Type 2 diabetes; FPG: 7.0–13.3 mmol/l or 2hPG: 11.1–22.9 mmol/l; age: 18–70 years; normal renal function	Diet and exercise advices were provided		*Baseline balance* statistically different in gender between groups No significant difference of FPG, PPG, HbA1c between the CHM and control groups after treatment	Side effects (CHM: n = 1)	Short study period; Not double blind trial
			*Wumei Wan* 3 packages daily, 12 weeks *Formulas* Huanglian, Huangbai, Ganjiang, Ginseng, Danggui, Huajiao, et al.	*Metformin* 500 mg/time, twice daily, 12 weeks			
Wu and Fan [34] China Oct 2012–Jan 2013	Type 2 diabetes	*Sample size* n = 152 *CHM group* n = 76; 48 males and 28 females; age: 48–66 years *Control group* n = 76; 35 males and 41 females; age: 47–68 years *Inclusion criteria* diabetes symptoms and any plasma glucose ≥ 11.1 mmol/l; FPG ≥ 7.0 mmol/l; 2hPG ≥ 11.1 mmol/l during OGTT	*Self-proposed Chinese herbal medicines with insulin* 1 dose daily, 2 weeks *Formulas* Guijianyu, Zhimu, Gegen, Jineijin, Zexie, Ginseng, et al.	*Insulin injection* Novolin 30R before breakfast and lunch, 2 weeks	*Baseline balance* Yes Significant more 20% decrease of insulin use in the CHM group than that in the control group after treatment; Significant less treatment days and frequency of hypoglycaemia in the CHM group than those in the control group after treatment	N/A	N/A
Cai et al. [35] China No information on study period	Type 2 diabetes	*Sample size* n = 67 *CHM group* n = 37 *Control group* n = 30 *Inclusion criteria* diabetes course < 5 years, fasting serum glucose > 7.0 mmol/l and/or 11.1 mmol/l after meal	Diet and exercise advices were provided		*Baseline balance* Yes Significantly decreased serum glucose and increased insulinogenic index in the CHM group before and after treatment; Significantly increased HDL in the CHM group than that in the placebo group after treatment	No side effects	Small sample size; Short follow-up
			*Lycium barbarum Poly-saccharide capsule* 300 mg/day, twice daily, 3 months *Formulas* Gouqi	*Placebo* 300 mg/day body weight, twice daily, 3 months			
Lian et al. [36] China Apr 2013–Oct 2013	Type 2 diabetes	*Sample size* n = 186 *CHM group* n = 92 *Control group* n = 94 *Inclusion criteria* diagnosed type 2 diabetes; standard diet control and exercise therapy; taking metformin in a steady dose for over 3 months; HbA1c ≥ 7.0%; FPG: 7.0–13.9 mmol/l or 2hPG ≥ 11.1 mmol/l; BMI: 18–40 kg/m^2^; age: 18–70 years	Diet and exercise advices were provided		*Baseline balance* Yes Significantly decreased HbA1c and increased HOMA-β in the CHM group before and after treatment; More significant decrease of HbA1c, FPG, 2hPG in the CHM group than those in the placebo group after treatment	N/A	Short study period; Small sample size
			*Jinlida with metformin* (1500 mg/kg/day) 1 granule/time, 3 times daily, 12 weeks *Formulas* Shuweicao, Yinyanghuo, Ginseng, Huangjing, Cangzhu, Kushen, et al.	*Placebo with metformin* (1500 mg/kg/day) 1 granule/time, 3 times daily, 12 weeks			
Zhang et al. [37] China Jan 2011–Dec 2013	Type 2 diabetes	*Sample size* n = 219; 112 males and 107 females; age: 38–74 years *CHM group* n = 109 *Control group* n = 110 *Inclusion criteria* diagnosed type 2 diabetes treated with insulin alone; FPG ≥ 7.0 mmol/l or 2hPG ≥ 11.1 mmol/l; age: 18 years and older; standard food containing100 g of carbohydrate during intervention	*Shen-Qi-Formula with insulin injection* (300 IU, twice daily before breakfast and dinner) 100 ml/time, 3 times daily, 12 weeks *Formulas* Shengdihuang, Huangqi, Zhidahuang, Ginseng, Shanzhuyu, Shuweicao, et al.	*Insulin injection* 300 IU, twice daily before breakfast and dinner, 12 weeks	*Baseline balance*: Yes Significantly decreased FPG, HbA1c in both CHM and control groups before and after treatment; Significantly decreased HOMA-IR and insulin usage level in the CHM group, while significantly increased insulin usage level in the control group, before and after treatment after treatment; More significant decrease of FPG, PPG, HbA1c in the CHM group than those in the control group after treatment	Transient hypoglycemia (Control: n = 1)	N/A
Hu et al. [38] China No information on study period	Type 2 diabetes	*Sample size* n = 112 *CHM group* n = 59 *Control group* n = 53 *Inclusion criteria* newly diagnosed type 2 diabetes (illness course ≤5 years); only taking metformin for treatment; age: 18–75 years; HbA1c: 6.5–9.0% despite taking two 500 mg metformin tablets daily	Diet and exercise advices were provided		*Baseline balance* Yes Significantly decreased FPG, HbA1c in both CHM and placebo groups before and after treatment; More significant decrease of FPG, HbA1c in the CHM group than those in the placebo group after treatment	No side effects	Small sample size; No group without lifestyle intervention; Almost 25% participants lost from both groups
			*Jianyutangkang tablet with Metformin* (1.5 g/time, 3 times daily) 3 tablets/time, 3 times daily, 26 weeks *Formulas* Ciwujia, Zhimu, Guijianyu	*Placebo with Metformin* 1.5 g/time, 3 times daily, 26 weeks			
Li et al. [39] China Jun 2014–Dec 2014	Type 2 diabetes	*Sample size* n = 38 *CHM group* n = 23 *Control group* n = 15 *Inclusion criteria* diagnosed Type 2 diabetes; not on a regimen of antidiabetic medical treatment at least 3 months before screening, or on a regimen of antidiabetic treatment no more than 3 months at any time in the past, or on a stable regimen of metformin monotherapy for at least 8 weeks; age:18–70 years; HbA1c: 7.0–10.0%; FPG ≤ 13 mmol/l; BMI: 19–30 kg/m^2^	During the intervention, metformin remains stable		*Baseline balance* Yes Significantly decreased 1 h and 2 h PPG, HbA1c in the CHM group before and after treatment without significant difference between these two groups after treatment; No significant difference of FPG between the CHM and control groups after treatment	Gastrointestinal side effects (lower in the CHM group than control group) Slightly higher liver and kidney function indices in the CHM group than those in the control group	Short study period; Small sample size; Missing data of BMI in follow-up period
			*Mulberry twig alkaloid tablet with Acarbose placebo* (50 mg/time, 3 times daily) 50 mg-100 mg/time, 3 times daily, 24 weeks *Formulas* Sangzhi	*Placebo with Acarbose* (50–100 mg/time, 3 times daily) 50 mg/time, 3 times daily, 24 weeks			
Wang et al. [40] China No information on study period	Hyperlipidemia	*Sample size* n = 446 *CHM group* n = 324; 188 males and 136 females; mean age: 56 years *Control group* n = 122; 73 males and 49 females; mean age: 56 years *Inclusion criteria* serum TC ≥ 5.95 mmol/l, LDL-C ≥ 3.41 mmol/l, or TG: 2.26-4.52 mmol/l; HDL-C ≤ 1.04 mmol/l (male)/1.16 mmol/l (female); no medication for hyperlipidemia for more than 4 weeks and received dietary advice for 2–4 weeks	During the intervention, no medications affecting serum lipids		*Baseline balance* Yes Significantly decreased TC, LDL-C, TG in both CHM and control groups before and after treatment; More significant decrease of TC, LDL-C, TG and increase of HDL-C in the CHM group than those that in the control group after treatment; Significant higher total effective rate in the CHM group (93.2%) than that in the control group (50.8%)	CHM group: Heartburn; flatulence; Dizziness; Exacerbation of preexisting stomachache	N/A
			*Monascus purpureus rice preparation* 3 tablets (600 mg)/time, twice daily, 8 weeks *Formulas* Red yeast rice	*Jiaogulan* 3 tablets (600 mg)/time, twice daily, 8 weeks *Formulas* Jiaogulan			
Yang et al. [41] China Feb 2002–May 2004	Hyperlipidemia	*Sample size* n = 96 *CHM group* n = 56; 31 males and 25 females; mean age: 69 years *Control group* n = 40; 29 males and 11 females; mean age: 68 years Both groups have cases with coronary heart disease, hypertension, and cerebral vascular disease *Inclusion criteria* TC > 5.7 mmol/l and/or TG > 1.7 mmol/l; TCM diagnosed for phlegm-damp and blood stasis syndrome	During the intervention, no other drugs		*Baseline balance* Yes Significantly decreased TC, LDL-C in both CHM and control groups before and after treatment; Significantly decreased TG in the CHM group before and after treatment; More significant decrease of TC, LDL-C in the CHM group than those in the control group after treatment	No side effects	N/A
			*Danshen Jueming granules* 24 g/time, twice daily *Formulas* Taizishen, Danshen, Juemingzi, Shanzha, Zexie, Chenpi, et al.	*Xuezhikang* *capsules* 0.8 g/time, 3 times daily			
Ai et al. [42] China No information on study period	Hyperlipidemia	*Sample size* n = 60 *CHM group* n = 30 *Control group* n = 30 *Inclusion criteria* BMI < 35 kg/m^2^; TC ≥ 5.72 mmol/l and TG > 4.52 mmol/l; age: 18 years and older	During the intervention, no other lipid-modulating drugs		*Baseline balance* statistically different in the serum TG level between groups Significantly decreased in the TC, LDL-C in both CHM and control groups before and after treatment; More significant decrease of TC, LDL-C in the control group than those in the CHM group after treatment	Diarrhea (CHM: n = 8); Myalgia and epigastric discomfort (Control: n = 2)	N/A
			*Daming capsule* 2 g/time, twice daily, 6 weeks *Formulas* Dahuang, Ginseng, Juemingzi, Danshen	*Pravastatin* 10 mg/time, once daily, 6 weeks			
Xu et al. [43] China No information on study period	Hyperlipidemia	*Sample size* n = 77 *CHM group* n = 37; 17 males and 20 females; mean age: 59 years *Control group* n = 40; 20 males and 20 females; mean age: 61 years *Inclusion criteria* TC ≥ 5.72 mmol/l or TG ≥ 1.70 mmol/l or HDL-C ≤ 1.04 mmol/l (male)/1.17 mmol/l (female); TCM diagnosed for phlegm-damp and blood stasis syndrome	During the intervention, no drugs affecting the blood lipid metabolism		*Baseline balance* Yes Significantly decreased TC, TG, LDL-C, BMI in the CHM group and significantly decreased LDL-C, BMI in the control group, before and after treatment; More significant decrease of TC, TG in the CHM group than those in the control group after treatment; Significantly lower recurrence rate in the CHM group than that in the control group after treatment	No side effects	N/A
			*Antihyperlipidemic decoction* 150 ml/time, twice daily, 8 weeks *Formulas*: Yiyiren, Shengpuhuang, Zexie, Shengshanzha, Huangqi, Juemingzi, et al.	*Zhinbiticose* 1050 mg/time, 3 times daily, 8 weeks			
Hu et al. [44] Hong Kong No information on study period	Hyperlipidemia	*Sample size* n = 40 *CHM group* n = 20; 6 males and 14 females; mean age: 58 years *Control group* n = 20; 10 males and 10 females; mean age: 55 years *Inclusion criteria* diagnosed dyslipidemia with lipid-lowering therapy or fasting LDL-C ≥ 4.1 mmol/l or TG ≥ 1.7 mmol/l; plasma LDL-C ≥ 2.6 mmol/l or ≥ 1.8 mmol/l for those with high cardiovascular risk following lipid-lowering treatment and diet or plasma TG ≥ 1.7 mmol/l following a lipid-lowering diet; age: 18 years and older	*A multiherb formula* 4 capsules in the morning and 4 capsules in the evening, 12 weeks *Formulas* Shanzha, Zexie, Yumixu, Sangye, Lingzhi, Heshouwu	*Placebo* 4 capsules in the morning and 4 capsules in the evening, 12 weeks	*Baseline balance* statistically different in the LDL-C level between groups More significant decrease of LDL-C in the CHM group than that in the placebo group after treatment; No significant difference of LDL-C in the CHM group before and after treatment	CHM group: n = 11, including one stomach upset; Placebo group: n = 12, including one acid reflux	Not balanced baseline data of the two groups; Small sample size; Lack of consideration of the different types of dyslipidemia
Moriarty et al. [45] USA and China Apr 2011–Aug 2012	Hyperlipidemia	*Sample size* n = 116 *CHM group 1* n = 36; 6 males and 30 females; mean age: 58 years *CHM group 2* n = 42; 13 males and 29 females; mean age: 56 years *Control group* n = 38; 11 males and 27 females; mean age: 56 years *Inclusion criteria* TC ≥ 13.3 mmol/l; LDL-C: 8.9-12.2 mmol/l; TG < 22.2 mmol/l; BMI < 36 kg/m^2^; age: 18 years and older	During the intervention, no lipid-lowering drugs, investigational agent, medications promoting weight loss, agents affecting lipid metabolism		*Baseline balance* Yes Significantly decreased LDL-C in both two CHM groups before and after treatment, without significant difference between these two groups after treatment; The total effective rates at about 48% of LDL-C by ≥30% in the two CHM groups before and after treatment, without significant difference between these two groups	CHM groups 1, 2: n = 5, not CHM-related side effects (thyroid cancer, pulmonary embolism, fractured leg) Placebo group: n = 3	Not representative data; More females than males; Short treatment period
			*CHM group 1: Xuezhikang* *1200* *mg* 2 capsules (300 mg) and 2 placebo daily, 12 weeks *CHM group 2:* *Xuezhikang 2400* *mg* 4 capsules (300 mg) daily, 12 weeks *Formulas* Red yeast rice	*Placebo* 4 placebo capsules daily, 12 weeks			
Heber et al. [46] USA No information on study period	Hyperlipidemia	*Sample size* n = 83; 46 males and 37 females; age: 34–78 years *Inclusion criteria* LDL-C > 4.14 mmol/l and TG < 2.94 mmol/l; no treatment for hypercholesterolemia before; normal liver and renal function	Diet advices were provided		*Baseline balance* Yes Significantly decreased TC, TG, LDL-C in the CHM group before and after treatment; More significant decrease of TC, LDL-C in the CHM group than those in the placebo group after treatment	Placebo group: Rash (n = 1); Headaches (n = 1); Concurrent development of pneumonia (n = 1)	N/A
			*Red yeast rice capsule* 1 capsule (600 mg), 2.4 g daily, 12 weeks *Formulas* Red yeast rice	*Rice powder placebo capsule* 1 capsule (600 mg), 2.4 g daily, 12 weeks			
Lin et al. [47] Taiwan Dec 2001–Jan 2003	Hyperlipidemia	*Sample size* n = 79 *CHM group* n = 39; 23 males and 16 females; mean age: 46 years *Control group* n = 40; 22 males and 18 females; mean age: 47 years *Inclusion criteria* TC ≥ 6.22 mmol/l; LDL-C ≥ 4.14 mmol/l; TG ≤ 4.52 mmol/l; age: 18–65 years; BMI < 30 kg/m^2^; no lipid-lowering drugs 4 weeks before screening	Diet advices were provided		*Baseline balance* Yes Significantly decreased TC, TG, LDL-C in the CHM group before and after treatment; More significant decrease of TC, TG, LDL-C in the CHM group than those in the placebo group after treatment	CHM group: Drug-related side effects (n = 6)	No record of diets of the participants
			*Monascus purpureus Went rice* 1 capsule (600 mg)/time, twice daily, 8 weeks *Formulas* Red yeast rice	*Rice powder placebo* 1 capsule (600 mg)/time, twice daily, 8 weeks			
Wei et al. [48] China Mar 2006–Sep 2007	Impaired glucose tolerance	*Sample size* n = 140 *CHM group* n = 70; 31 males and 39 females; mean age: 51 years *Control group* n = 70; 32 males and 38 females; mean age: 51 years *Inclusion criteria* 2hPG: 7.8–11.1 mmol/l; age: 25–70 years; BMI: 18.5–35.0 kg/m^2^; no IGT treatment before; TCM diagnosed for spleen-stomach dampness-heat syndrome	*Tang No.1 granule with IGT knowledge education* 2 packets/time, twice daily, 6 months *Formulas*: Dangshen, Fushen, Huangqi, Shanyao, Huangqin, Huanglian, et al.	*IGT knowledge education*	*Baseline balance* Yes Significantly decreased FPG, 2hPG, HbA1c, TG, HOMA-IR in the CHM group before and after treatment;More significant decrease of FPG, 2hPG, HbA1c, TG, HOMA-IR in the CHM group than those in the control group after treatment;More patients with IGT reversed to normal in the CHM group (19.1%) than that in the control group (3.1%)	No side effects	N/A
Gao et al. [49] China No information on study period	Impaired glucose tolerance	*Sample size* n = 510 *CHM group* n = 255; 110 males and 145 females; mean age: 49 years *Control group* n = 255; 112 males and 143 females; mean age: 51 years *Inclusion criteria* 2hPG: 7.8–11.1 mmol/l after OGTT and FPG > 7.0 mmol/l; age: 25–75 years; BMI: 20–35 kg/m^2^	Co-administered medications: calcium antagonists, α blockers or ACE antagonists, or β-blockers or thiazide for hypertension control		*Baseline balance* Yes Significantly decreased 2hPG, HbA1c, BMI, FIN, HOMA-IR in the CHM group before and after treatment; More significant decrease of FPG, 2hPG, HbA1c, FIN, HOMA-IR in the CHM group than those in the control group after treatment; More patients with IGT reversed to normal in the CHM group (29.1%) than those in the control group (13.6%) after treatment; Lower risk of IGT patients progressing to Type 2 diabetes in the CHM group (22.2%) than that in the placebo group (43.9%)	Mild abdominal distension (CHM: n = 4; Control: n = 3)	Small sample size; Short follow-up
			*Tangzhiping granule with Standard health care advice* 5 g/time, twice daily, 5 days a week *Formulas* Huanglian, Sangbaipi, Gegen	*Standard health care advice*			
Fang et al. [50] China No information on study period	Impaired glucose tolerance	*Sample size* n = 514 *CHM group* n = 257; 136 males and 121 females; mean age: 55 years *Control group* n = 257; 142 males and 115 females; mean age: 55 years *Inclusion criteria* 2hPG: 7.8–11.1 mmol/l and FPG < 7.0 mmol/l; TCM diagnosed for spleen deficiency and dampness syndrome; age: 25–70 years; no IGT treatment before; no participation in clinical trials within the 3 months before screening	*Shenzhu Tiaopi granule with lifestyle intervention* 8.8 g/time, twice daily, 12 months *Formulas* N/A	*Lifestyle intervention*	*Baseline balance* Yes More patients with IGT reversed to normal in the CHM group (42.2%) than that in the control group (32.9%); Lower risk of IGT patients progressing to Type 2 diabetes in the CHM group (8.5%) than that in the placebo group (15.3%)	CHM group: n = 9 Placebo group: n = 5 Gastrointestinal reactions were the most common side effects	Short follow-up; No consensus about efficacy of the CHM approach
Lian et al. [51] China Aug 2008–Mar 2010	Impaired glucose tolerance	*Sample size* n = 420 *CHM group* n = 210; 98 males and 112 females; mean age: 53 years *Control group* n = 210; 106 males and 104 females; mean age: 52 years *Inclusion criteria* 2hPG: 7.8–11.1 mmol/l after OGTT and FPG > 7.0 mmol/l; age: 25–70 years; no IGT treatment before; no participation in clinical trials within the 3 months before screening	Diet and exercise advices were provided		*Baseline balance* Yes More patients with IGT reversed to normal in the CHM group (63.1%) than that in the control group (46.6%); Lower risk of IGT patients progressing to Type 2 diabetes in the CHM group (18.2%) than that in the placebo group (29.3%)	CHM group: n = 15 Placebo group: n = 11 Gastrointestinal reactions were the most common side effects	Short study period; No data on plasma insulin and HbA1c; Small sample size
			*Tianqi capsule* 5 capsules/time, 3 times daily, 12 months *Formulas* Huangqi, Nvzhenzi, Huanglian, Tianhuafen, Shihu, Jixueteng, et al.	*Placebo* 5 capsules/time, 3 times daily, 12 months			
Huang et al. [52] China Mar 2013–Jul 2015	Impaired glucose tolerance	*Sample size* n = 120 *CHM group* n = 60; 31 males and 29 females; mean age: 52 years *Control group* n = 60; 35 males and 25 females; mean age: 51 years *Inclusion criteria* 2hPG: 7.8–11.1 mmol/l and FPG < 7.0 mmol/l; age: 30–70 years; no diabetes history; normal blood test, urine, stool, liver and renal function	*Tangyiping granules with lifestyle intervention* 10 g/time, twice daily, 12 weeks *Formulas* Huangqi, Baishao, Huanglian, Danshen, Banxia, Gegen	*Lifestyle intervention*	*Baseline balance* Yes Significantly decreased 2hPG, HbA1c, HOMA-IR, TG in the CHM group before and after treatment; More significant decrease of 2hPG, HbA1c, HOMA-IR, TG in the CHM group than those in the control group after treatment; More patients with IGT reversed to normal in the CHM group (58.3%) than that in the control group (26.7%); Lower risk of IGT patients progressing to Type 2 diabetes in the CHM group (16.7%) than that in the placebo group (31.7%)	No severe side effects	Small sample size; Short follow-up; Insufficient outcome measures
Shi et al. [53] China Apr 2014–Oct 2014	Impaired glucose tolerance	*Sample size* n = 61 *CHM group* n = 32; 17 males and 15 females; mean age: 47 years *Control group* n = 29; 14 males and 15 females; mean age: 50 years *Inclusion criteria* 2hPG: 7.8–11.1 mmol/l after OGTT and FPG < 7.0 mmol/l; age: 20–80 years; BMI: 18–30 kg/m^2^	Diet, exercise, smoking/alcohol consumption advices were provided; no other CHM products with similar function		*Baseline balance* Yes Significantly decreased FPG, 2hPG, HbA1c, HOMA-IR, BMI in the CHM group before and after treatment; More significant decrease of HbA1c, 2hPG, HOMA-IR in the CHM group than those in the control group after treatment; Lower risk of IGT patients progressing to Type 2 diabetes in the CHM group (6.2%) than that in the placebo group (17.2%); More patients with IGT reversed to normal in the CHM group (43.8%) than that in the control group (6.9%)	Gastrointestinal reactions (n = 2)	Short study period; Small sample size
			*Jinlida granule* 1 granule (9 g)/time, 3 times daily, 12 weeks *Formulas* Ginseng, Fuling, Cangzhu, Gegen, Huangjing, Zhimu, et al.	*No drug intervention*			
Grant et al. [54] Australia Jun 2007–Dec 2009	Impaired glucose tolerance	*Sample size* n = 71 *CHM group* n = 39; 15 males and 24 females; mean age: 58 years *Control group* n = 32; 18 males and 14 females; mean age: 60 years *Inclusion criteria* FPG < 7.0 mmol/l and 2hPG: 7.8–11.0 mmol/l; age: 18 years and older	*Jiangtang Xiaozhi* 3 capsules/time, 3 times daily, 16 weeks *Formulas* Nvzhenzi, Huangqi, Huanglian, Kunbu, Lizhihe, Jianghuang	*Placebo* 3 capsules/time, 3 times daily, 16 weeks	*Baseline balance* Yes More significant decrease of fasting insulin, HDL in the CHM group than those in the placebo group after treatment; No information on the efficacy of CHM before and after treatment	CHM group: moderate dizziness (n = 1)	Short study period; Small sample size
Pan et al. [55] China Jul 2003–Aug 2003	Obesity	*Sample size* n = 78 *CHM group* n = 40; 18 males and 22 females; mean age: 41 years *Control group* n = 38; 17 males and 21 females; mean age: 41 years *Inclusion criteria* BMI ≥ 25 kg/m^2^; age: 20–50 years	*Dietary powder* 1 package (9 g)/time, twice daily, 7 weeks *Formulas* Lotus rhizome, Green tea, Sanqi	*Placebo* 1 package (9 g)/time, twice daily, 7 weeks	*Baseline balance* Yes Significantly decreased body mass, percentage of body fat, BMI, WC, HC in the CHM group before and after treatment; More significant decrease of body mass, percentage of body fat, BMI, WC, HC in the CHM group than those in the placebo group	Irritability (CHM: n = 1; Placebo: n = 1); Nausea (CHM: n = 2; Placebo: n = 1); Constipation (Placebo: n = 2)	N/A
Zhou et al. [56] China May 2010–Feb 2011	Obesity	*Sample size* n = 134 *CHM group* n = 70; 31 males and 39 females; mean age: 40 years *Control group* n = 64; 29 males and 35 females; mean age: 40 years *Inclusion criteria* BMI: 28–40 kg/m^2^; WC ≥ 85 cm (male)/80 cm (female); age: 18–60 years; TCM diagnosed for qi and phlegm stasis syndrome	*Xin-Ju-Xiao-Gao-Fang (full-dose)* 170 ml decoction/time, twice daily, 24 weeks *Formulas* Dahuang, Zhishi, Huanglian, Juemingzi	*Xin-Ju-Xiao-Gao-Fang (10% of full-dose)* 170 mL decoction/time, twice daily, 24 weeks	*Baseline balance* Yes More significant decrease of body weight, WC, HC, FIN in the CHM group than those in the control group after treatment	Minor side effects (e.g. skin rash) (CHM: n = 4; Control: n = 3)	Short study period; No follow-up; No true placebo group
Lenon et al. [57] Australia No information on study period	Obesity	*Sample size* n = 117 *CHM group* n = 59; 10 males and 49 females; mean age: 39 years *Control group* n = 58; 10 males and 48 females; mean age: 40 years *Inclusion criteria* BMI ≥ 30 kg/m^2^; age: 18–60 years	During the intervention, no other medications for obesity management		*Baseline balance* Yes Significantly decreased body weight, BMI, body fat in the CHM group and increased body weight, BMI, body fat in the placebo group, before and after treatment; More significant decrease of body weight, BMI in the CHM group than those in the placebo group after treatment	Nausea (CHM: n = 4) Headache (CHM: n = 9) Decrease of appetite (Placebo: n = 2)	N/A
			*Chinese herbal medicine formula RCM-104* 4 capsules/time, 3 times daily, 12 weeks *Formulas* Green tea, Juemingzi, Huaihua	*Placebo* 4 capsules/time, 3 times daily, 12 weeks			
Hioki et al. [58] Japan No information on study period	Obesity and impaired glucose tolerance	*Sample size* n = 81; mean age: 54 years *CHM group* n = 41 *Control group* n = 40 *Inclusion criteria* FPG < 7.0 mmol/l and 2hPG: 7.8–11.1 mmol/l after OGTT	Diet and exercise advices were provided		*Baseline balance* Yes Significantly decreased body weight, WC, HC, TC,TG, LDL-C in both CHM and placebo groups before and after treatment; Significantly decreased fasting insulin, HOMA-IR in the CHM group before and after treatment; More significant decrease of WC in the CHM group than that in the placebo group after treatment	CHM group: Loose bowels (n = 3)	N/A
			*Bofu-tsusho-san* 3 times daily, 24 weeks *Formulas* Jingjie, Bohe, Shigao, Gancao, Lianqiao, Mahuang, et al.	*Placebo* 3 times daily, 24 weeks			
Gao & Hu [59] China No information on study period	Type 2 diabetes and hyperlipidemia	*Sample size* n = 80 *CHM group* n = 40; 22 males and 18 females; mean age: 59 years *Control group* n = 40; 20 males and 20 females; mean age: 59 years *Inclusion criteria* FPG > 7.0 mmol/l and blood PG > 6.1 mmol/l	During the intervention, hypoglycemic agents remain stable		*Baseline balance* Yes Significantly decreased TC, TG, LDL-C and increased HDL-C in the CHM group before and after treatment, without significant difference compared to the control group after treatment	Control group: Slight elevation of ALT (n = 2)	N/A
			*Taizhi’an capsule with Simvastatin* (10 mg daily) 0.9 g/time, 3 times daily, 12 weeks *Formulas* N/A	*Simvastatin* 20 mg daily, 12 weeks			
Poppel et al. [60] Netherlands May 2012–Mar 2013	Hyperlipidemia and hypertension	*Sample size* n = 20; 14 males and 6 females; mean age: 58 years *CHM group* n = 9 *Control group* n = 11 *Inclusion criteria* fasting LDL-C > 3.5 mmol/l and/or TG > 1.7 mmol/l; age: 40–70 years; SBP > 140 mmHg and/or DBP > 90 mmHg despite taking antihypertensive drugs	*Danshen capsules* 4 capsules (500 mg)/time, 3 time daily, 4 weeks *Formulas* Danshen	*Placebo* 4 capsules (500 mg)/time, 3 time daily, 4 weeks	*Baseline balance* Yes Significantly increased LDL-C in the CHM group before and after treatment, without significant difference compared to the placebo group; No significant difference of BP between the CHM and placebo groups after treatment after treatment	CHM group: Headache (n = 5); Dizziness (n = 3); Change in stool frequency (n = 3); Flatulence (n = 2); Peripheral facial nerve paralysis (n = 1)	Carry-over effect
Chu et al. [61] China Jan 2008–Dec 2009	Metabolic syndrome	*Sample size* n = 90 *CHM group* n = 60; 28 males and 32 females; mean age: 51 years *Control group* n = 30; 13 males and 17 females; mean age: 50 years *Inclusion criteria* diagnosed central obesity; WC > 90 cm (male)/80 cm (female) and/or BMI > 25 kg/m^2^; fasting blood glucose ≥ 6.1 mmol/l and/or 2hPG ≥ 7.8 mmol/l or having diabetes history; TG > 1.7 mmol/l and/or HDL-C < 0.9 mmol/l(male)/1.0 mmol/l (female); age: 18–70 years	Diet and exercise advices were provided; During the intervention, no other CHM with hypoglycemic, lipid-lowering and antihypertensive effects		*Baseline balance* Yes Significantly decreased BMI, waist-to-hip ratio, TC, TG, LDL-C, 2hPG and increased HDL-C in the CHM group before and after treatment; More significant decrease of BMI, TC, LDL-C, 2hPG and increase of HDL-C in the CHM group than those in the placebo group after treatment	CHM group: Diarrhea (n = 1)	N/A
			*Pu’er tea extract capsules* 4 capsules/time, twice daily, 3 months *Formulas* Pu’er tea	*Placebo* 4 capsules/time, twice daily, 3 months			
Chen et al. [62] China Oct 2011–Oct 2012	Hypertension and metabolic syndrome	*Sample size* n = 43 *CHM group* n = 22; 14 males and 8 females; mean age: 49 years *Control group* n = 21; 14 males and 7 females; mean age: 49 years *Inclusion criteria* diagnosed metabolic syndrome; average BP > 135/85 mmHg when awake and > 120/75 mmHg during sleep or SBP ≥ 140 mmHg and/or DBP ≥ 90 mmHg; age: 18–65 years	Diet and exercise intervention were provided		*Baseline balance* Yes Significantly decreased body weight, WC, BMI, FPG, 2hPG, FIN, HOMA-IR, SBP, DBP, daytime SBP, daytime DBP, nighttime SBP in the CHM group before and after treatment; More significant decrease of WC, waist-to-hip ratio, 2hPG, HOMA-IR, FIN, SBP, DBP, daytime SBP and DBP than those in the placebo group after treatment	CHM group: Skin allergy (n = 2)	N/A
			*Yiqi* *Huaju formula* 1 bag/time, twice daily, 12 weeks *Formulas* Huangqi, Zexie, Huanglian, Yinchen, Puhuang	*Placebo* 12 weeks			
Azushima et al. [63] Japan Jun 2010–Mar 2013	Hypertension and obesity	*Sample size* n = 106 *CHM group* n = 54; 28 males and 26 females; mean age: 59 years *Control group* n = 52; 29 males and 23 females; mean age: 60 years *Inclusion criteria* diagnosed hypertension with a history of antihypertensive treatment more than 4 weeks; BMI > 25 kg/m^2^; age: 20–79 years	Diet and exercise advices were provided		*Baseline balance* Yes Significantly decreased daytime SBP, daytime DBP, body weight, BMI in the CHM group before and after treatment; More significant decrease of daytime SBP, body weight, BMI in the CHM group than those in the control group after treatment	CHM group: Gastric irritation (n = 1); Constipation (n = 1); Elevation of serum hepatic enzyme level (n = 1)	Not a double-blinded placebo-controlled study; Short study period
			*Bofu-tsusho-san with Antihypertensive therapy* 2.5 g/time, once daily, 24 weeks *Formulas* Jingjie, Bohe, Shigao, Mahuang, Gancao, Lianqiao, et al.	*Antihypertensive therapy* No further information			

*2hPG* 2-hour postprandial glucose, *BP* blood pressure, *BMI* body mass index, *DBP* diastolic blood pressure, *FIN* fasting plasma insulin, *FPG* Fasting plasma glucose, *HbA1c* glycated hemoglobin, *HC* hip circumferences, *HDL* high-density lipoprotein, *HDL-C* high-density lipoprotein cholesterol, *HOMA-β* homeostatic model assessment β-cell function, *HOMA-IR* homeostatic model assessment insulin resistance, *IGT* Impaired glucose tolerance, *LDL-C* low-density lipoprotein cholesterol, *LVMI* left ventricular mass index, *MBP* mean blood pressure, *OGTT* oral glucose tolerance test, *PPG* postprandial plasma glucose, *SBP* systolic blood pressure, *TC* total cholesterol, *TG* triglyceride, *TO* original heart rate, *WC* waist circumference

### Quality assessment

Two authors independently assessed the methodological quality of the included studies using the Cochrane risk of bias criteria [17]. The characteristics of RCTs that might be related to selection bias (random sequence generation and allocation concealment), performance bias (blinding of participants and personnel), detection bias (blinding of outcome assessment), attrition bias (incomplete outcome data), reporting bias (selective outcome reporting), and other bias were evaluated. Disagreements regarding the risks of bias of some studies were resolved through discussion amongst these two authors (Table 3).

**Table 3:** Risk of bias assessment of the included studies using the Cochrane risk of bias tool

Author, Country, Publication year	Stroke risk factor	Random sequence generation	Allocation concealment	Blinding of participants and personnel	Blinding of outcome assessment	Incomplete outcome data	Selective reporting	Other bias
Lin et al. [18], China, 2004	(Primary) Hypertension	Unclear	Unclear	High risk	Unclear	Low risk	Unclear	Unclear
Li [19], China, 2005	(Primary) Hypertension	Unclear	Unclear	High risk	Unclear	Unclear	Unclear	Unclear
Ye et al. [20], China, 2009	(Primary) Hypertension	Unclear	Unclear	Low risk	Low risk	Unclear	Low risk	Unclear
Zhao et al. [21], China, 2010	(Primary) Hypertension	Unclear	Unclear	Low risk	Unclear	Unclear	Unclear	Unclear
Zhong et al. [22], China, 2011	(Primary) Hypertension	Low risk	High risk	High risk	Unclear	Low risk	Low risk	Unclear
Yang et al. [23], Taiwan, 2012	(Uncontrolled primary) Hypertension	Low risk	Unclear	High risk	Low risk	Unclear	Low risk	High risk
Tong et al. [24], China, 2013	Hypertension	Low risk	High risk	High risk	Low risk	Unclear	Low risk	Unclear
Wu et al. [25], China, 2014	(Primary) Hypertension	Low risk	Low risk	Unclear	Low risk	Low risk	Low risk	Unclear
Li et al. [26], China, 2010	(Isolated systolic) Hypertension	Low risk	Unclear	Low risk	Unclear	High risk	Unclear	Unclear
Chen et al. [27], China, 2012	(Polarized) Hypertension	Low risk	Unclear	High risk	Unclear	Unclear	Unclear	High risk
Gong et al. [28], China, 2010	Hypertension with cardiac damage	Unclear	Unclear	High risk	Unclear	Low risk	Unclear	Unclear
Xu et al. [29], China, 2013	Hypertension, hypertension with diabetes, hypertension with coronary heart disease	Unclear	Unclear	High risk	Unclear	Unclear	Low risk	High risk
Chao et al. [30], China, 2009	Type 2 diabetes	Low risk	Low risk	Low risk	Low risk	Low risk	Low risk	Unclear
Ji et al. [31], China, 2013	Type 2 diabetes	Low risk	Low risk	High risk	Low risk	Low risk	Low risk	Unclear
Tong et al. [32], China, 2013	Type 2 diabetes	Low risk	Unclear	Low risk	Low risk	High risk	Unclear	Unclear
Tu et al. [33], China, 2013	Type 2 diabetes	Low risk	Low risk	High risk	Unclear	Low risk	Low risk	Unclear
Wu & Fan [34], China, 2014	Type 2 diabetes	Unclear	Unclear	High risk	Unclear	Unclear	Unclear	Unclear
Cai et al. [35], China, 2015	Type 2 diabetes	Low risk	Unclear	Low risk	Unclear	Low risk	Low risk	Unclear
Lian et al. [36], China, 2015	Type 2 diabetes	Low risk	Low risk	Low risk	Low risk	Low risk	Low risk	Unclear
Zhang et al. [37], China, 2015	Type 2 diabetes	Low risk	Low risk	High risk	Unclear	Unclear	Low risk	Unclear
Hu et al. [38], China, 2016	Type 2 diabetes	Low risk	High risk	Low risk	Low risk	High risk	Low risk	Unclear
Li et al. [39], China, 2016	Type 2 diabetes	Low risk	Low risk	Low risk	Low risk	Low risk	Low risk	Unclear
Wang et al. [40], China, 1997	Hyperlipidemia	Low risk	Unclear	High risk	Low risk	Low risk	Low risk	Unclear
Yang et al. [41], China, 2006	Hyperlipemia	Unclear	Unclear	Unclear	Unclear	Unclear	Unclear	High risk
Ai et al. [42], China, 2009	Hyperlipemia	High risk	High risk	High risk	High risk	Unclear	Low risk	High risk
Xu et al. [43], China, 2009	Hyperlipemia	Unclear	Unclear	High risk	Unclear	Unclear	Unclear	Unclear
Hu et al. [44], Hong Kong, 2014	Hyperlipemia	Low risk	Low risk	Low risk	Unclear	Low risk	Low risk	High risk
Moriarty et al. [45], USA & China, 2014	Hyperlipemia	Low risk	Low risk	Low risk	Unclear	Low risk	Low risk	Unclear
Heber et al. [46], USA, 1999	Hyperlipidemia	Unclear	Unclear	Low risk	Unclear	Low risk	Low risk	High risk
Lin et al. [47], Taiwan, 2005	Hyperlipidemia	High risk	Unclear	Low risk	Low risk	Low risk	Low risk	High risk
Wei et al. [48], China, 2008	Impaired glucose tolerance	High risk	Unclear	High risk	Unclear	Low risk	Unclear	Unclear
Gao et al. [49], China, 2013	Impaired glucose tolerance	Low risk	Unclear	High risk	Unclear	Low risk	Low risk	Unclear
Fang et al. [50], China, 2014	Impaired glucose tolerance	Unclear	Unclear	High risk	Unclear	Low risk	Low risk	Unclear
Lian et al. [51], China, 2014	Impaired glucose tolerance	Low risk	Low risk	Low risk	Low risk	Low risk	Low risk	Unclear
Huang et al. [52], China, 2016	Impaired glucose tolerance	Low risk	Low risk	High risk	Unclear	Low risk	Low risk	Unclear
Shi et al. [53], China, 2016	Impaired glucose tolerance	Low risk	Unclear	High risk	Unclear	High risk	Low risk	Unclear
Grant et al. [54], Australia, 2013	Impaired glucose tolerance	Low risk	Low risk	Low risk	Low risk	Low risk	Unclear	High risk
Pan et al. [55], China, 2005	Obesity	Low risk	Unclear	Low risk	Unclear	Low risk	Unclear	High risk
Zhou et al. [56], China, 2014	Obesity	Low risk	Unclear	Low risk	Unclear	Unclear	Low risk	Unclear
Lenon et al. [57], Australia, 2012	Obesity	Unclear	Low risk	Low risk	Unclear	Low risk	Low risk	Unclear
Hioki et al. [58], Japan, 2004	Obesity and impaired glucose tolerance	Low risk	High risk	Low risk	Unclear	Unclear	Low risk	High risk
Gao & Hu [59], China, 2006	Type 2 diabetes and hyperlipidemia	Unclear	Unclear	High risk	Unclear	Low risk	Low risk	Unclear
Poppel et al. [60], Netherlands, 2015	Hyperlipidemia and hypertension	High risk	Unclear	Low risk	Unclear	Low risk	Low risk	High risk
Chu et al. [61], China, 2011	Metabolic syndrome	High risk	Unclear	Low risk	Unclear	Low risk	Low risk	Unclear
Chen et al. [62], China, 2013	Hypertension and metabolic syndrome	Low risk	Unclear	Low risk	Unclear	High risk	Low risk	Unclear
Azushima et al. [63], Japan, 2015	Hypertension and obesity	Low risk	Unclear	High risk	High risk	Low risk	Low risk	High risk

## Results

**Fig. 1:**
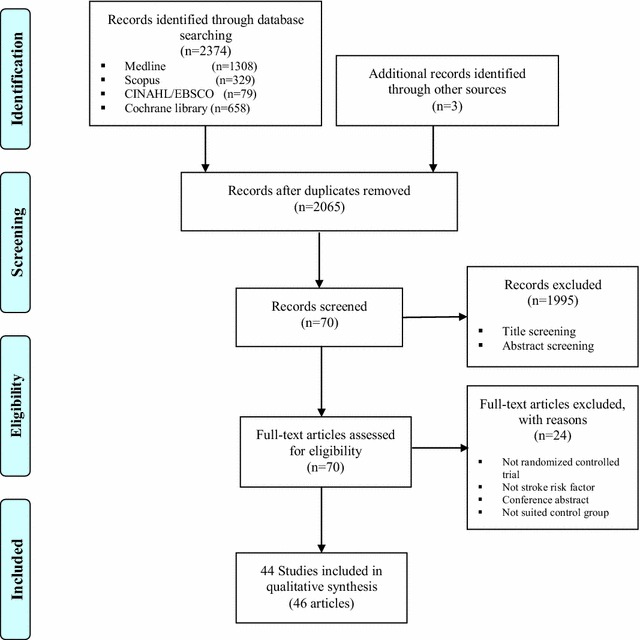
PRISMA flowchart of literature search and study selection

The systematic review reported in this paper has been registered on the PROSPERO (International prospective register of systematic reviews, #CRD42017060107). The PRISMA flowchart of literature search and study/article selection has been shown in Fig. 1. A total of 2377 papers were identified (2374 via database searches and three additional papers via Google Scholar). After removing duplicates, a total of 2065 papers remained for review. From amongst these, 70 manuscripts were identified for full review following title and abstract screening. Further screening of the full texts identified 46 publications (reporting on 46 RCTs) as eligible for final inclusion in the systematic review. Twelve of the included articles report on the efficacy of CHM for hypertension (1340 participants), 10 for diabetes (2004 participants), eight for hyperlipidaemia (997 participants), seven for IGT (1805 participants), three for obesity (329 participants), and six for the combination of several stroke risk factors (420 participants). No manuscript reported on a trial investigating the efficacy of CHM interventions for the stroke risk factor of transient ischemic attack or atrial fibrillation as a primary outcome. The characteristics of included studies with regards to the CHM interventions for hypertension, diabetes, hyperlipidaemia, IGT, obesity, and combined stroke risk factors are summarized in Table 2.

### Hypertension

Eight RCTs were focused upon primary (essential) hypertension [18–25], one with isolated systolic [26], one with elder polarized hypertension [27], and two with hypertension and related cardiovascular diseases [28, 29]. Of the 12 RCTs on CHM for hypertension, 11 RCTs originated from China [18–22, 24–29]. Amongst the hypertension-focused RCTs, one RCT compared ‘CHM, biomedicine plus lifestyle’ intervention with ‘biomedicine plus lifestyle’ intervention [27] and showed significant decreased systolic blood pressure (SBP) before and after treatment of both intervention groups and a similar effect on controlling SBP between these two groups after treatment. Another two RCTs compared two different CHM interventions using different inclusion criteria of people with hypertension  [19, 21]—these studies both reported a significant decrease of SBP and diastolic blood pressure (DBP) via all the CHM interventions examined with higher effective rate of treatments in the CHM groups than those in the control groups. Another three RCTs compared ‘CHM’ interventions with ‘biomedicine’ interventions and employed consistent inclusion criteria regarding SBP (140–179 mmHg) and DBP (90–109 mmHg) of participants, reporting a statistically significant decrease of SBP and DBP before and after treatment of both groups and a similar effect on controlling SBP and DBP between these two groups after treatment [18, 22, 24]. Another six RCTs compared ‘CHM plus biomedicine’ interventions with ‘biomedicine alone’ or ‘biomedicine plus placebo’ interventions [20, 23, 25, 26, 28, 29]. It is noteworthy that two of these six trials  [20, 28] examined the efficacy of the same CHM products (Xuezhikang capsule) at different dose levels, demonstrating a significant decrease of SBP and DBP before and after treatment of both intervention groups and a silimar effect on SBP and DBP control between these two groups after treatment. Also amongst these six RCTs, three were three-armed RCTs which compared either ‘CHM plus biomedicine’ intervention versus ‘biomedicine/no intervention’, ‘CHM’ interventions versus ‘CHM plus biomedicine’ or ‘placebo plus biomedicine’ intervention, or two types of preparations of a ‘CHM plus biomedicine’ intervention versus ‘placebo plus biomedicine’ intervention [25, 26, 28], showing inconsistent findings with regards to the decrease of SBP or DBP amongst the three groups after treatment. *Gouteng* (钩藤) [18, 19, 21, 24, 25, 29] and *Tianma* (天麻) [18, 22, 25–27] were the most frequently used Chinese herbs in the hypertension-focused RCTs included, and all the CHM interventions using *Gouteng* and/or *Tianma* reported significant pre-post effectiveness regarding the decrease of SBP (and/or DBP) level. Also, *Gouteng* was the principal CHM formula constituent amongst four out of six hypertension-focused RCTs presenting between-group effectiveness of the investigated CHM interventions on the decrease of SBP (and/or DBP) levels compared to control interventions [21, 24, 25, 29]. In addition, the sample size of hypertension-focused RCTs ranged from 55 to 219. Six hypertension-focused RCTs did not provide the age and gender profile of the participants in either CHM group or control group [20, 22, 23, 26, 27, 29]. The duration of the hypertension-focused trials ranged from 2 weeks to 24 months, with the majority of trials conducted between 4 and 12 weeks.

Eight hypertension-focused RCTs reported safety-related information and no deaths were noted [18, 19, 21, 23, 26–29]. One trial reported five cases of serious side effects of the ‘CHM plus biomedicine’ intervention group [29]. One trial (sample: 55) reported 13 mild side effects in the ‘CHM plus biomedicine’ intervention group and 15 in the ‘placebo plus biomedicine’ control group [23]. Only two of the papers reporting results from hypertension-focused RCTs listed any study limitations including small sample size and short study period [23, 24]. As for risk of bias in the hypertension-focused RCTs, three papers provided information on the allocation concealment [22, 24, 25] and four on the blinding of outcome assessment [20, 23–25]. Additionally, only three trials reported double-blinding of participants and personnel involved [20, 21, 26].

### Diabetes

All of the 10 included diabetes-focused RCTs were focusing upon patients diagnosed with Type 2 diabetes mellitus and all these RCTs were conducted in China [30–39]. Amongst the 10 RCTs examining the efficacy of CHM on controlling the glucose level of patients with diabetes, four RCTs compared ‘CHM’ intervention to ‘placebo’ [32], ‘CHM plus biomedicine’ intervention to ‘placebo plus biomedicine’ intervention [39], and further, ‘CHM plus lifestyle’ intervention to ‘placebo plus lifestyle’ intervention [30, 35]. These four trials indicated more significant decreased glucose level [e.g. fasting plasma glucose (FPG), 2-hour postprandial glucose (2hPG), glycated hemoglobin (HbA1c)] by using CHM products when compared to the placebos after treatment, while this significant between-group variance in the decrease of glucose level showed no statistical significance when both CHM interventions and placebos were used concurrently with biomedicine or lifestyle intervention. Also amongst these 10 diabetes-focused RCTs, ‘CHM plus biomedicine’ intervention was compared to ‘biomedicine’ intervention, showing a more significant decrease of insulin usage by the CHM plus biomedicine treatment after treatment [34]. Also, after treatment, ‘CHM, biomedicine plus lifestyle’ interventions were found to achieve a more significant decrease of FPG, HbA1c, or hypoglycemia when compared to either ‘biomedicine plus lifestyle’ intervention [31, 37] or ‘placebo, biomedicine plus lifestyle’ intervention [36, 38]. Of the nine diabetes-focused RCTs providing CHM formulas, *Huanglian* (黄连) was the most common Chinese herb [30, 32–34, 36], followed by *Ginseng* (人参) [33, 34, 36, 37], *Shanzhuyu* (山茱萸) [34, 36, 37], *Dahuang* (大黄) [32, 34, 37], and *Huangqi* (黄芪) [30, 34, 37]. The CHM interventions examined in three out of five diabetes-focused RCTs, showing significant between-group effectiveness on the decrease of glucose level, indicated that the combination of these five commonly used Chinese herbs played a vital role for the efficacy of type 2 diabetes management [34, 36, 37]. All diabetes-focused RCTs defined inclusion criteria of diabetes based on different FPG, 2hPG, and/or HbA1c levels, and all the tested CHM products used in these RCTs were different. The sample size of the diabetes-focused RCTs ranged from 43 to 627. Only one RCT provided the age and gender profile of participants in the CHM and control groups [35]. The duration of the trials ranged from 2 weeks to 12 months, with the majority of trials conducted between 3–12 months.

Only two diabetes-focused RCTs failed to report safety-related information and no death were noted [34, 36]. The side effects of CHM products reported in the diabetes-focused RCTs are generally moderate, such as constipation, gastrointestinal disorders, and urinary tract infection. However, three diabetes-focused RCTs showed that CHM interventions caused slightly abnormal liver and kidney function after 3, 6, and 12 months, respectively  [31, 32, 39]. Six diabetes-focused RCTs have specified their study limitations, with a short study period being the most common issue, followed by small sample size and no/short follow-up period [32, 33, 35, 36, 38, 39]. As for risk of bias of the diabetes-focused RCTs, one trial failed to use the random sequence generation method [34], three trials did not report information on allocation concealment [32, 34, 35], four trials failed to apply a double-blinding method [31, 33, 34, 37], and four trials did not provide details on the blinding outcome assessment [33–35, 37].

### Hyperlipidemia

Half of the eight RCTs on CHM for the treatment of hyperlipidemia originated from China [40–43]. Amongst the hyperlipidemia-focused RCTs, two compared ‘CHM’ interventions with ‘biomedicine’ interventions [42, 43], two compared different ‘CHM’ interventions [40, 41], two compared ‘CHM’ interventions with ‘placebos’ [44, 45] and two compared ‘CHM plus lifestyle’ interventions with ‘placebo plus lifestyle’ interventions [46, 47]. Although the inclusion criteria of people with hyperlipidemia shown in the included hyperlipidemia-focused RCTs are limited to the total cholesterol (TC), triglyceride (TG), low-density lipoprotein cholesterol (LDL-C), high-density lipoprotein cholesterol (HDL-C), and/or body mass index (BMI) levels, the threshold value of these indices are diverse across the RCTs. It is worth noting that Monascus purpureus rice preparation (Xuezhikang capsule in Chinese) of which the main ingredient is red yeast rice, was tested in four hyperlipidemia-focused RCTs [40, 45–47]. The effects of the red yeast rice products are not consistent across these four RCTs. When the ‘red yeast rice product plus lifestyle’ intervention was compared with ‘placebo plus lifestyle’ intervention, a more significant decrease of TC and LDL-C was found in the red yeast rice product group after treatment. However, there was no significant improvement in TC or LDL-C amongst those receiving the red yeast rice product alone when compared to placebo alone. Amongst the rest four hyperlipidemia-focused RCTs, *Danshen* (丹参) [41–43], *Juemingzi* (决明子) [41–43], *Zexie* (泽泻) [41, 43, 44], and/or *Shanzha* (山楂) [41, 43, 44] were the main constituents of the CHM formulas examined and three of these trials reported the significant between-group effectiveness of the investigated CHM interventions on the decrease of TC, LDL-C, and/or TG levels [41, 43, 44] compared to control interventions. The sample size of the hyperlipidemia-focused RCTs ranged from 40 to 446. Only two hyperlipidemia-focused RCTs did not provide the age and gender profile of the participants in CHM and control groups [42, 46]. The duration of the trials ranged from 6 weeks to 12 months while one trial did not specify the study period.

All hyperlipidemia-focused RCTs reported safety-related information and no deaths were noted. Three trials specified their side effects in the CHM intervention groups, including heartburn/flatulence [40], diarrhea [42], and stomach upset [40, 44]. Three hyperlipidemia-focused RCTs reported their study limitations including small sample size, lack of balanced baseline data between the CHM and control groups and no record of the participants’ dietary control  [44, 45, 47]. As for risk of bias of the hyperlipidemia-focused RCTs, five trials did not use the random sequence generation method [41–43, 46, 47], only two trials specified the appropriate allocation concealment [44, 45], and six trials failed to employ the blinding of outcome assessment [41–46].

### Impaired glucose tolerance

The seven RCTs on CHM for the treatment of IGT originated from China (n = 6) [48–53] and Australia (n = 1) [54]. Amongst the IGT-focused RCTs, one compared ‘CHM’ with ‘placebo’ [54], five compared ‘CHM plus lifestyle’ interventions with ‘lifestyle’ interventions alone [48–50, 52, 53], and one compared ‘CHM plus lifestyle’ intervention with ‘placebo plus lifestyle’ intervention [51]. The inclusion criteria regarding the 2hPG level remain stable (7.8–11.0 mmol/l) while the FPG level is either <7.0 or >7.0 mmol/l across all the IGT-focused RCTs. Additionally, all the tested CHM products within the IGT-focused RCTs are different. Despite the variation in the inclusion criteria and CHM products, the results on the effects of CHM interventions are consistent throughout all IGT-focused trials. Specifically, more people with IGT reversed to normal in the CHM group (range 19.1–63.1%) compared to those in the control group (range 3.1–46.6%) and less people with IGT progressed to Type 2 diabetes in the CHM group (range 6.2–22.2%) compared to those in the control group (range 15.3–43.9%). Of the six IGT-focused RCTs with detailed CHM formulas, five reported the significant between-group effectiveness of the investigated CHM interventions regarding the decrease of FPG, 2hPG, and/or HbA1c levels compared to control interventions [48, 49, 52–54] and *Huanglian* (黄连) and *Gegen* (葛根) were the only Chinese herbs both included in these five IGT-focused trials. The sample size of the IGT-focused RCTs ranged from 61 to 514, and all these RCTs provided the age and gender profile of participants in the CHM and control groups (897 males, 939 females, mean age 53 years with the range from 47 to 60 years). The duration of the IGT-focused trials ranged from 3 to 12 months.

All IGT-focused RCTs reported safety-related information and no deaths were noted. The most common side effects reported in the CHM groups were dizziness, gastrointestinal reactions, and abdominal distension. Almost all IGT-related RCTs provided information on their study limitations including a short study period and short follow-up period as well as small sample size. As for risk of bias of the IGT-focused RCTs, three trials provided information about the allocation concealment [51, 52, 54], two trials provided details on the blinding of outcome assessment [51, 54], and two trials reported double-blinding of participants and personnel [51, 54].

### Obesity

Two RCTs on CHM for the treatment of obesity originated from China [55, 56] and one from Australia [57]. The three obesity-focused trials compared three different CHM products with their placebos. BMI is the key indicator of the inclusion criteria of all obesity-focused RCTs included. However, the threshold value of BMI was set differently across these trials. Amongst the obesity-focused RCTs, CHM products all showed more decrease of body weight than placebos after treatment. *Green tea* (绿茶) [55, 57] and *Juemingzi* (决明子) [56, 57] were the Chinese herbs included in two CHM formulas amongst these three obesity-focused trials. The sample size of the obesity-focused RCTs ranged from 78 to 134 and all these RCTs provided the age and gender profile of participants in the CHM and placebo groups. There were 115 males and 214 females across all the obesity-focused RCTs with a mean age of 40 years, ranging from 39 to 41 years. The duration of the obesity-focused trials ranged from 7 weeks to 6 months.

All obesity-focused RCTs reported safety-related information and no death were noted. CHM interventions were reported more side effects than the placebos, including nausea, headache, and skin rash. One obesity-focused RCT indicated the study limitations including short study period, no follow-up period, and no true placebo group [56]. As for risk of bias of the obesity-focused RCTs, all trials reported the double-blinding of participants and personnel while these trials failed to provide any details of the blinding of outcome assessment.

### Combined stroke risk factors

Six RCTs exploring the efficacy of CHM on one or more of the stroke risk factors were identified in the systematic review. Specifically, one trial examined the ‘CHM plus lifestyle’ intervention for the treatment of ‘IGT and obesity’ compared to ‘placebo plus lifestyle’ intervention, showing significant efficacy on both IGT and obesity before and after treatment and a significant effect on obesity control between groups after treatment [58]; Two trials examined the ‘CHM plus biomedicine’ interventions for the treatment of ‘diabetes and hyperlipidemia’ and ‘hypertension and hyperlipidemia’ compared to the ‘biomedicine’ intervention [59] and ‘placebo plus biomedicine’ intervention [60], respectively—both of these studies found similar effect on the combined stroke risk factors between groups after treatment. Moreover, three trials examined the ‘CHM, biomedicine plus lifestyle’ interventions for the treatment of ‘metabolic syndrome’ [61], ‘hypertension and metabolic syndrome’ [62], and ‘hypertension and obesity’ [63] compared to the ‘biomedicine plus lifestyle’ interventions with or without placebo, respectively, indicating significant effects on all included stroke risk factors by the CHM interventions compared to the control groups after treatment. Except the *Bofu*-*tsusho*-*san* (防风通圣散) used in two trials, all the other CHM interventions involved exploring a combination of multiple stroke risk factors were different and therefore it is unable to report the commonly used Chinese herbs which are vital for the efficacy of combined stroke risk factors across these six RCTs. The sample size of the RCTs focused upon combined stroke risk factors ranged from 20 to 106, and two of these RCTs failed to provide the age and gender profile of participants in the CHM and control groups [58, 60]. The duration of the RCTs exploring the combined stroke risk factors ranged from 4 to 6 months.

All RCTs focusing upon combined stroke risk factors reported safety-related information and no deaths were noted. Five out of these six RCTs reported that side effects only occurred in the CHM group [58, 60–63] including headache, dizziness, gastrointestinal reactions, and skin allergy. Only two RCTs focusing upon combined stroke risk factors identified their study limitations [60, 63], including failure to double-blind the RCT, short study period and carry-over effect. As for risk of bias of the RCTs focusing upon combined stroke risk factors, no trial reported appropriate allocation concealment and blinding of outcome assessment, and two trials were found to have a high risk of bias regarding the random sequence generation [60, 61].

## Discussion

This paper reports the first comprehensive systematic review of the literature concerning the use of CHM amongst people at greatest risk(s) of stroke. A number of significant findings from our review are important for future evidence-based planning and priority setting for research in stroke prevention.

Our analyses show some positive efficacy and safety evidence of varied CHM interventions in lowering high blood pressure, high blood glucose, high cholesterol, high body BMI and a combination of multiple stroke risk factors. Importantly, our findings indicate that, compared to biomedicine alone/lifestyle modification alone/biomedicine plus lifestyle intervention, CHM monotherapy may be not sufficient enough for people to obtain their treatment goals when treating hypertension, diabetes, and hyperlipidemia, while an intervention of CHM as a supplement to biomedicine and/or a lifestyle intervention is more effective in lowering the levels of SBP/DBP, glucose, BMI, TC, 2hPG, and/or HbA1c. These findings from our review are in line with previous systematic reviews on CHM for cardiovascular diseases [12–14]. In addition, the evidence reported in the papers included with regards to the successful reversion from elevated blood glucose level to normal by using CHM interventions suggests that some CHM products, in combination with a lifestyle intervention, could be considered a potential effective therapeutic regimen for IGT, and these findings are consistent with a Cochrane review on CHM for IGT published in 2009 [13]. Although many RCTs identified in our review demonstrate the therapeutic benefits of CHM in people with a number of stroke risk factors, there is a lack of replicable evidence on CHM use in combined stroke risk factors. It is worth noting that a CHM product (red yeast rice preparation), a medicinal food  [64], has been used several times not only for the management of hypertension but also for hyperlipidemia. However, the control interventions of all RCTs examining the efficacy of this rice preparation are different. Therefore, no trial included in our review paper has tested exactly the same CHM and control interventions for the treatment of any stroke risk factor(s).

Our findings show a large variation in the sample size and study period across the included RCTs. The potential risks of bias have been reported in the domains of allocation concealment, the blinding of participants and personnel, and/or the blinding of outcome assessment in the included RCTs. Most included trials have reported their safety information. No serious adverse events were noted although some studies showed some moderate side effects in the CHM groups.

Stroke risk factors vary by ethnic groups and such disparities may influence the etiology of stroke and the implementation of stroke prevention programs [65]. Nevertheless, the majority of studies on CHM use for stroke risk factors included in this review were conducted in China on Chinese populations. As such, the results shown in our review paper may not always be directly applicable to populations at risk of stroke in other countries beyond China. Furthermore, CHM is often composed of a number of herbs and is prescribed based on the unique Chinese medicine theory—syndrome differentiation. The replicability of these trial designs without Chinese medicine practitioners is therefore difficult.

There are some limitations to our systematic review that should be mentioned. Generalisability of the results from this systematic review is limited. Meanwhile, the overall ‘unclear’ reporting of research methodology in the included RCTs may limit the quality of the results reported in this review. In addition, our review was restricted to English peer-reviewed journal articles.

## Conclusion

Although the findings in this systematic review with regards to the effect of CHM for stroke modifiable risk factors should be interpreted with caution, the potential therapeutic benefits of CHM as a treatment—particularly in combination with biomedicine and/or lifestyle intervention—for different stroke risk factors needs to be further examined by conducting rigorous trials. Future research should be designed and implemented with adequate sample size, detailed reporting of the allocation concealment method, sufficient application of double-blinding with an adequate placebo and blinding of outcome assessment, and long-term follow-up in different countries. Moreover, it is important for future research on this topic to pay attention to potential drug-herb interactions as a major safety issue in trial design when participants need to take one or more co-administered biomedicine as well as CHM products.

## Abbreviations

2hPG: 2-hour postprandial glucose
BMI: body mass index
BP: blood pressure
CHM: Chinese herbal medicine
DBP: diastolic blood pressure
FIN: fasting plasma insulin
FPG: fasting plasma glucose
HbA1c: glycated hemoglobin
HC: hip circumferences
HDL: high-density lipoprotein
HDL-C: high-density lipoprotein cholesterol
HOMA-β: homeostatic model assessment β-cell function
HOMA-IR: homeostatic model assessment insulin resistance
IGT: impaired glucose tolerance
LDL-C: low-density lipoprotein cholesterol
LVMI: left ventricular mass index
MBP: mean blood pressure
OGTT: oral glucose tolerance test
PPG: postprandial plasma glucose
RCT: randomized controlled trial
SBP: systolic blood pressure
TC: total cholesterol
TG: triglyceride
TIA: transient ischaemic attack
TO: original heart rate
WC: waist circumference
